# Possible non-linear relation between prostate specific antigen and vitamin D: a machine learning study based on cross-section data

**DOI:** 10.7150/jca.96052

**Published:** 2024-05-13

**Authors:** Jia Shi, Chunyan Yin, Jinyi Wu

**Affiliations:** 1 Department of public health, Wuhan Fourth Hospital, Qiaokou, Wuhan, 430022, China.; 2 Department of medical Records and Statistics, Union Hospital, Tongji Medical College, Huazhong University of Science and Technology, Jianghan, Wuhan, 430022, China.; 3 School of public health, Fudan University, Xuhui, Shanghai, 200023, China.

**Keywords:** Vitamin D, 25(OH)D, prostate specific antigen, prostate cancer, NHANES.

## Abstract

**Objective:** Due to inconsistent results in earlier investigations regarding the relationship between vitamin D and prostate-specific antigen (PSA), this study was conducted to gain a deeper understanding of the association between vitamin D and PSA.

**Methods:** A total of 7174 male samples with 25(OH)D, PSA, and other variables were obtained from the National Health and Nutrition Examination Survey (NHANES) database. Three models, created through stepwise logistic regression, were employed to examine the dose-response association between PSA and 25(OH)D. Subsequently, restricted cubic spline analysis (RCS) was used to explore the nonlinear association between 25(OH)D and PSA. The study also compared the performance of four machine learning models in predicting PSA levels.

**Results:** The dose-response relationship indicated a negative impact of high 25(OH)D levels on PSA (p for trend 0.05). The odds ratio (OR) of Q4 (7.73 with 95% CI (0.26, 15.76)) was significantly higher than Q1 (6.23 with 95% CI (0.24, 12.57)). OR values in Q2 and Q3 were less than 1 (Q2= 0.57 with 95% CI (-6.37, 8.04) and Q3= 0.26 with 95% CI (-5.94, 6.86)), suggesting a potential protective effect of 25(OH)D on PSA. RCS analysis revealed a U-shaped relationship between blood 25(OH)D levels and PSA, with serum 25(OH)D in the range of 20-134 ng/ml showing a potential decrease in PSA levels. Above this range, an increase in 25(OH)D might elevate PSA levels. Age (2.67 with 95% CI 2.24 to 3.1) and BMI (17.52 with 95% CI 7.65 to 26.32), along with the OR of obesity (10.36 with 95% CI 0.68 to 20.18), were identified as potential PSA risk factors. Among the machine learning models, the random forest algorithm performed the best in predicting PSA levels.

**Conclusion:** This study revealed a U-shaped relationship between 25(OH)D and PSA, with PSA potentially declining when 25(OH)D is between 20 and 134 ng/mL and possibly rising above this range. The random forest method proved effective in both predicting PSA levels and guiding vitamin D dosage.

## Introduction

Prostate cancer stands as the third most prevalent cancer globally and holds the unfortunate position of being the most common malignancy among men, following lung and colon tumors. In 2017 alone, an estimated 161,360 new cases of prostate cancer and 26,730 related deaths were reported in the United States 1-3. The advent of serum PSA screening and the subsequent development of prostate biopsy since the late 1980s have notably increased the confirmed cases of prostate cancer 4, 5.

Earlier epidemiological investigations established a link between 25(OH)D and prostate cancer risk, particularly in studies involving African American men. These studies revealed lower 25(OH)D3 levels in this population, correlating with a significantly higher risk of prostate cancer and mortality compared to Caucasian men 6. The potential role of 25(OH)D compounds in cancer's etiology and treatment has been explored since the early 1970s. Hamster models exhibited cancer development inhibition with 25(OH)D compound treatment, and human cancer cells showed the presence of the 25(OH)D receptor (VDR), associated with *in vitro* growth arrest 7-10.

However, controlled studies demonstrated that high-dose 25(OH)D supplementation did not confer benefits to prostate cancer patients, as it increased the proportion of PSA responses. Large prospective studies further challenged the hypothesis that higher circulating 25(OH)D concentrations were associated with a reduced risk of prostate cancer. Surprisingly, elevated 25(OH)D levels might even be linked to an increased risk of aggressive disease 11-13.

The conflicting perspectives on the relationship between 25(OH)D and prostate cancer warranted further investigation. It was hypothesized that the relationship might not be linear, possibly following a U-shaped curve. This study, based on a comprehensive epidemiological survey from the United States Center for Disease Control and Prevention (CDC), included a sample of over 7000 males. The primary aim was to analyze the relationship between 25(OH)D and PSA, providing valuable insights to enhance our understanding of these factors and elucidating the threshold effects of 25(OH)D on PSA.

## Methods

### Dataset

**Figure 1:**
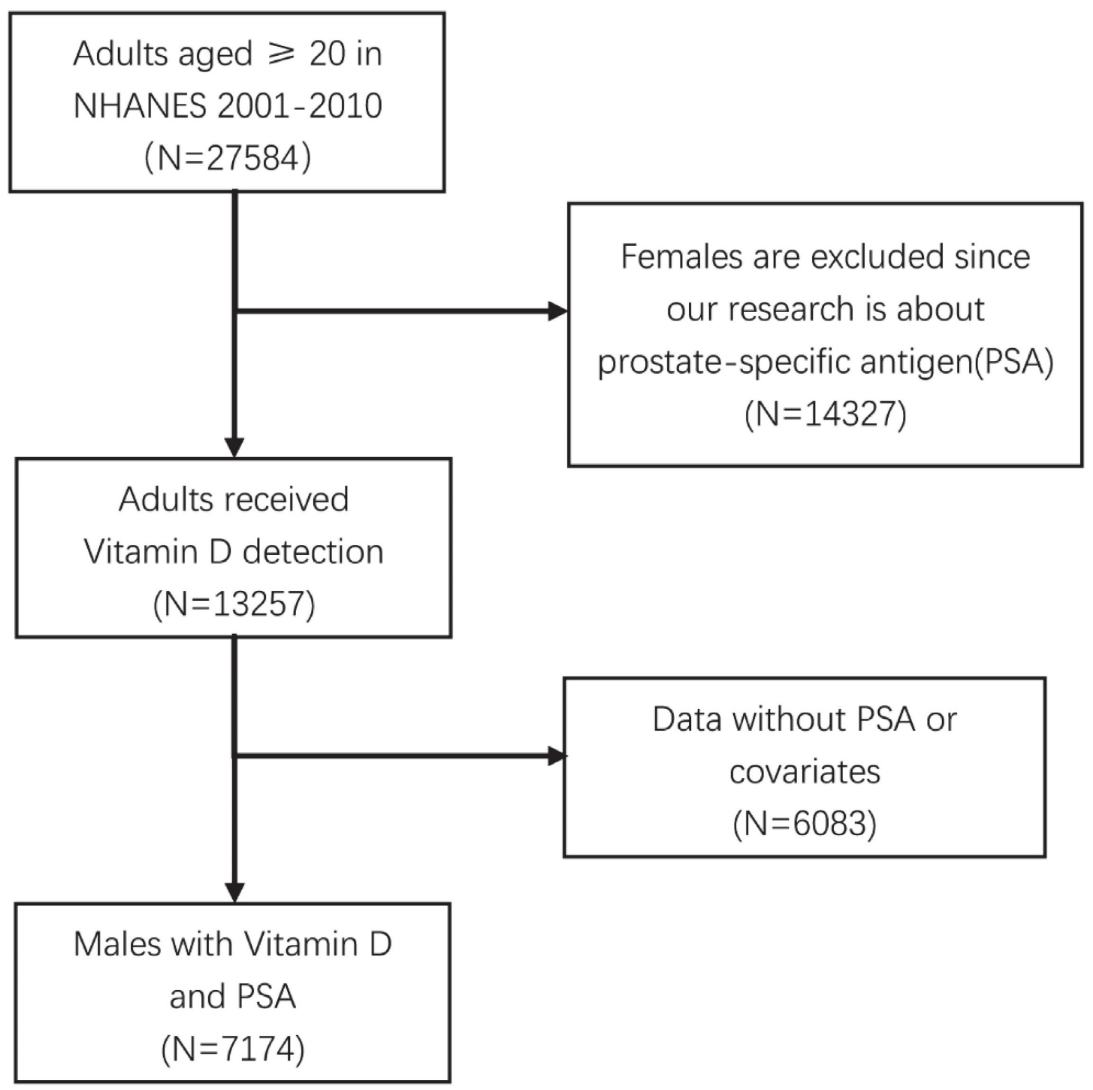
Data screening of eligible NHANES dataset.

The National Health and Nutrition Examination Survey (NHANES) program is designed to evaluate the health and nutritional status of both adults and children in the United States. To conduct this study, we accessed the NHANES database through the official CDC website at www.cdc.gov/nchs/nhanes/ 14-17. The dataset specifically focused on 25(OH)D and PSA, and the data acquisition process is illustrated in Figure 1. The inclusion samples should be more than 20 years old with eligible covariates.

Considering that PSA data were available only for the years 2001 to 2010, we acquired the original dataset comprising 27,584 cases of adults aged 20 years and older during this period. To refine our analysis, we excluded females and individuals who had not undergone testing for PSA or 25(OH)D. Following these criteria, we arrived at a final dataset comprising 7,174 eligible samples. The National Center for Health Statistics Ethics Review Board of the U.S. CDC authorized the NHANES methods, and all participants provided written informed consent. The study is based on a STROBE guideline (Strengthening the Reporting of Observational studies in Epidemiology).

### Covariates

To investigate the relationship between 25(OH)D and PSA, we meticulously curated data from the NHANES database spanning the years 2001-2010. Our dataset includes various demographic and health-related variables such as age, race, education, marital status, alcohol and tobacco consumption, BMI (Body Mass Index), PIR (Poverty Income Ratio), and the prevalence of hypertension and diabetes in the sample population.

Given that data on 25(OH)D were reported exclusively for the period 2001-2010, our analysis was focused on this timeframe. Additionally, since PSA is specific to males, our included sample comprises exclusively male individuals. Notably, for the sake of consistency, the measurement unit for both 25(OH)D and PSA in this study is ng/mL.

The dataset is enriched with information crucial to our analysis, providing a comprehensive overview of the selected sample. This includes not only the primary variables of interest, 25(OH)D and PSA, but also demographic factors, lifestyle choices, and health indicators, allowing for a robust exploration of potential correlations.

Recognizing the significance of NHANES sampling based on weights, we ensured the credibility of our analysis by considering laboratory quality assurance and monitoring conducted by Mobile Examination Centers (MECs). Specifically, we chose 'wtmec4yr' as the analytical weight, aiming to account for the complex sampling design and provide results that are representative of the broader population.

### Logistic regression and RCS

To explore the potential dose-response relationship between 25(OH)D and PSA, various covariates were considered in our analysis using stepwise logistic regression. Three distinct models were formulated: model 1= No adjustment, Model 2 = as Model 1 plus adjusted for sex, age (years, continuous), age squared, education (less than high school, high school graduate, some college and above), race (non-Hispanic white, non-Hispanic black, Mexican American, other), self-reported alcohol status (Yes and No) and self-reported smoking status (Yes and No) and Model 3 = Model 2 plus adjusted for BMI, self-reported hypertension (Yes and No) and self-reported diabetes (Yes and No).

The multifactorial logistic regression allows us to understand the nuanced effects of each covariate on PSA levels, providing a comprehensive perspective on the interplay between 25(OH)D and PSA with due consideration of various influencing factors.

Acknowledging the potential nonlinear nature of the relationship between 25(OH)D and PSA, we employed the Restricted Cubic Spline (RCS) technique for a comprehensive analysis. This involved constructing a plot to visually depict the intricate relationship between these two factors. RCS is a powerful regression technique utilizing piecewise polynomials of degree three (cubic splines) to model non-linear relationships between a response variable (PSA) and a predictor variable (25(OH)D). Unlike traditional linear regression, RCS does not assume a linear relationship. Instead, it employs cubic splines to estimate a smooth curve that best captures the underlying non-linear patterns in the data. Unlike traditional linear regression, RCS does not assume a linear relationship between the predictor and the response variable. Instead, it estimates a smooth curve that best fits the data points. The plot resulting from the RCS analysis will offer an insightful visualization of the dynamic relationship between 25(OH)D and PSA, allowing for a nuanced understanding of potential inflection points or trends that may not be apparent in linear models.

After an in-depth analysis of the relationship between 25(OH)D and PSA, our objective shifted towards predicting PSA levels. To achieve this, we employed four robust algorithms renowned for their strong predictive capabilities: Random Forest, Support Vector Machine (SVM), Logit Regression, and XGBoost.

To compare the predictive performance of these algorithms, we evaluated key indices for each model. Given that PSA is a continuous variable, we employed a scatter plot to analyze the consistency between real and predicted values. A 45° linear trend in the scatter plot indicates a high consistency, signifying good prediction efficacy. Given its superior prediction efficacy, our focus was primarily on understanding the principles underlying the Random Forest algorithm. This machine learning approach excels by leveraging multiple subsamples and constructing a forest of trees, leading to more accurate predictions and classifications.

## Results

In order to delve deeper into the relationship between PSA levels and various factors, quartile cut-off groupings were applied to the selected population. The analysis involved examining baseline data for each quartile group individually. Table 1 provides a snapshot of the distinct characteristics observed in the four groups.

A stepwise logistic regression (Table 2) was performed to analyze whether PSA increased as 25(OH)D increased in the Q2-Q4 group, using the Q1 group as a reference. It could be seen that in model 1, when only vitaminD-25(OH)D was used as the independent variable, there was a dose-response relationship between 25(OH)D and PSA (p for trend<0.05), and the OR of Q4 was much greater than 1 (6.23(0.24, 12.57)), which mean that with high levels of 25(OH)D, the higher the level of 25(OH)D, the PSA would rise correspondingly. While in Q2 and Q3 groups OR, values were less than 1 in model 1 (Q2 = -1.39 (-8.29, 6.02) and Q3 = 0.44 (-5.24, 6.46)), suggesting a possible protective effect of 25(OH)D on PSA at low levels of 25(OH)D.

After incorporating demographic and lifestyle data as covariates, model 2 was constructed and a dose-response relationship still existed. Finally, model 3 was constructed after incorporating BMI and disease history into the model, and also had a dose-response relationship. This was sufficient to suggest that the dose-response relationship between 25(OH)D and PSA was relatively stable.

**Figure 2:**
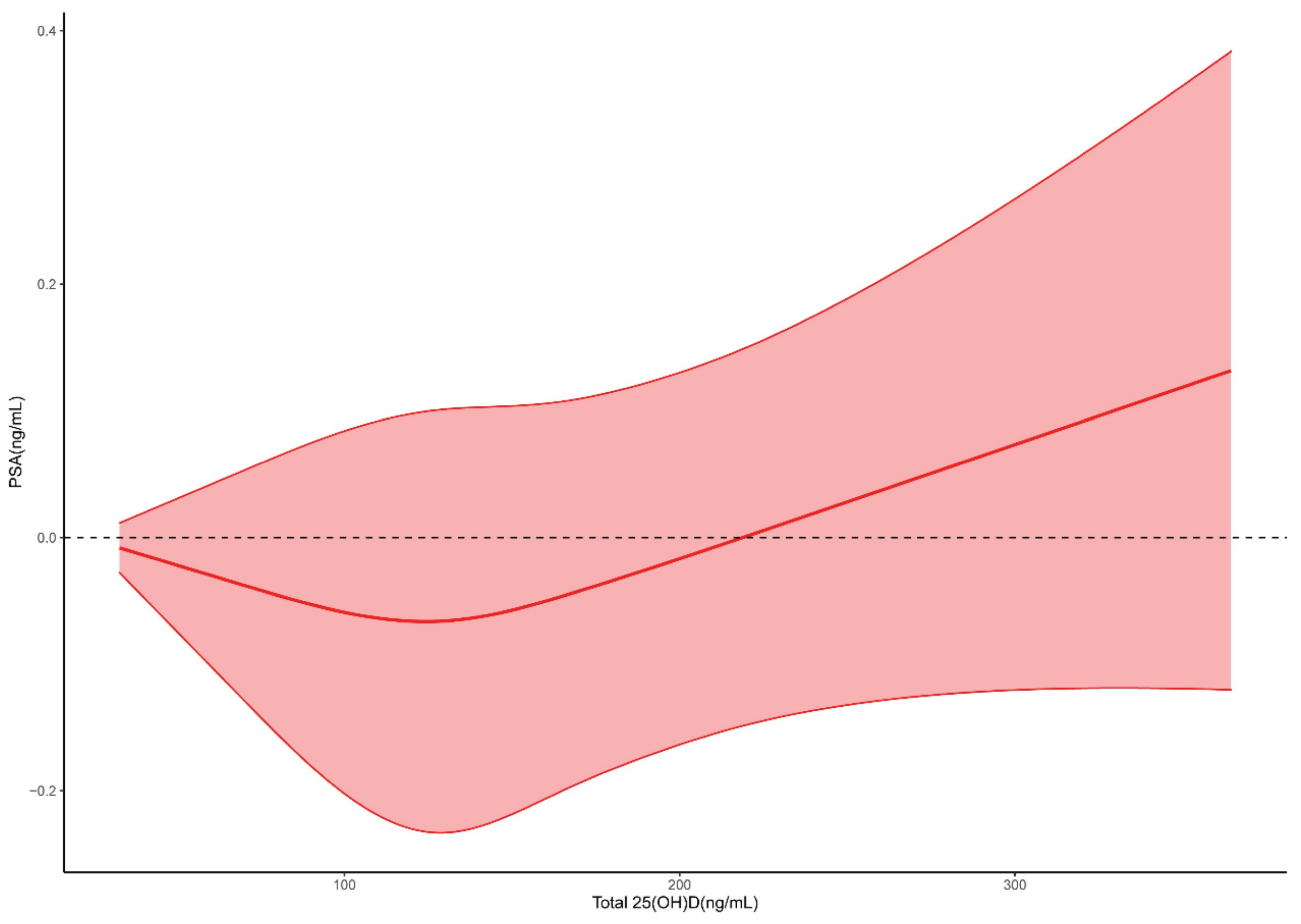
Restricted cubic spline for 25(OH)D and PSA.

By the results of logistic regression, we determined that there was a dose-response relationship between 25(OH)D and PSA, but it was not known whether the relationship was linear or nonlinear. So, we performed RCS analysis to observe the pattern of relationship between 25(OH)D and PSA. Figure 2 showed that blood 25(OH)D levels had a U-shape association with PSA, indicating that at low doses 25(OH)D supplementation reduced PSA, while beyond a certain threshold, continuous 25(OH)D supplementation lead to an increase in PSA. If serum 25(OH)D was in the range of 20-134 ng/mL, and an increase in 25(OH)D might reduce PSA levels, while beyond this range, an increase in 25(OH)D might lead to an incline in PSA levels.

After clarifying the dose-response relationship between 25(OH)D and PSA, we further analyzed the relationship between each covariate and PSA. Table 3 showed a correlation between age, BMI and PSA levels, where the OR of age was 2.67 with 95%CI (2.24, 3.1), indicating that PSA increases with age, and with BMI (OR of overweight=17.52 with 95%CI (7.65, 26.32), OR of obesity=10.36 with 95%CI (0.68, 20.18)).

**Figure 3:**
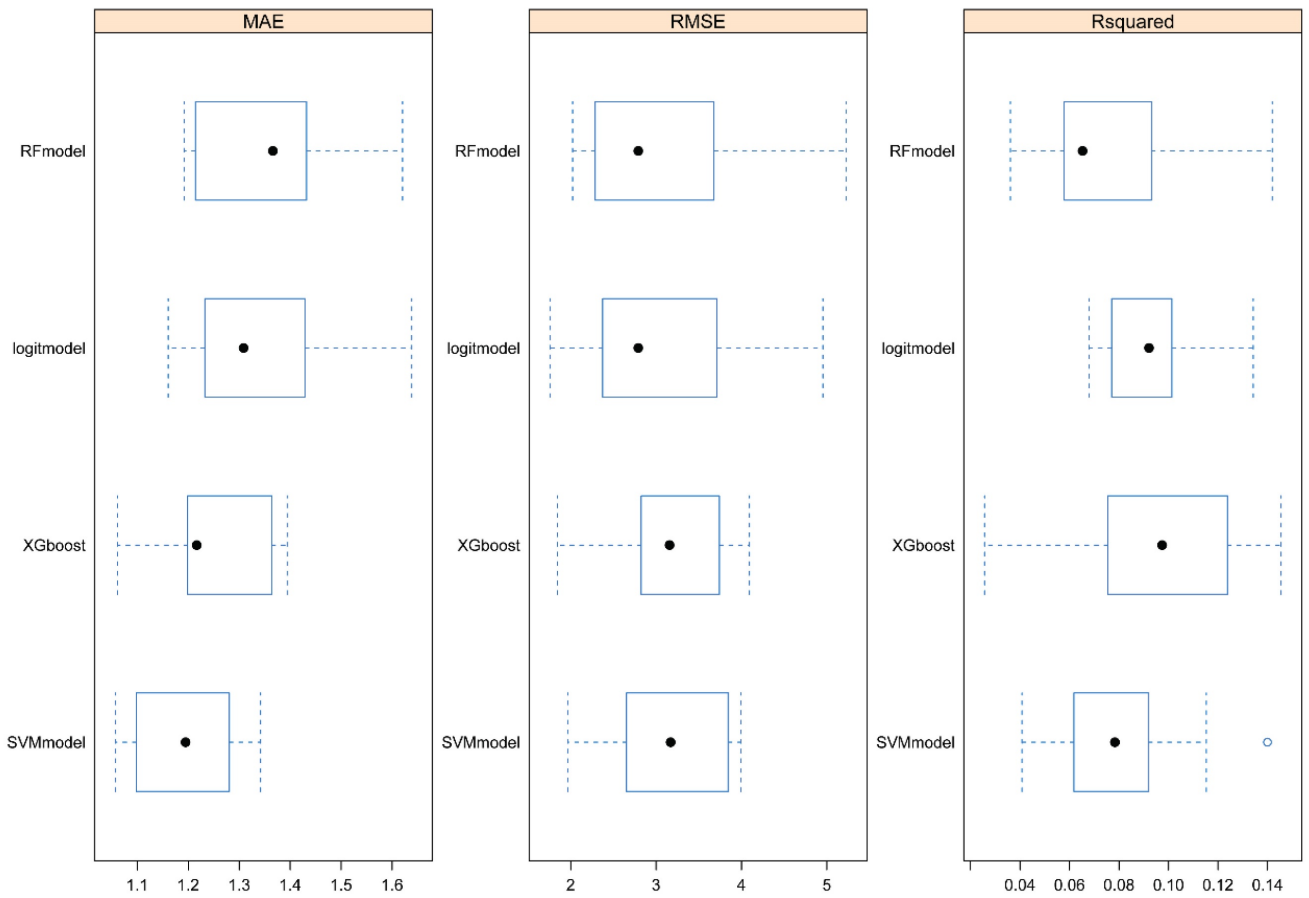
Evaluation of four machine learning algorithms. RFmodel: Random forest. Logitmodel: Logistic Regression. SVMmodel: Support vector machine.

To further investigate the relationship between 25(OH)D and PSA, we used four machine learning algorithms to predict the level of PSA. After the model was trained, predictions were made and the effects of the four algorithms were compared. From Figure 3, we could see that RF had the largest RMSE and R^2^ and the smallest MAE, indicating that the RF model fitted well and had good predictive ability.

**Figure 4:**
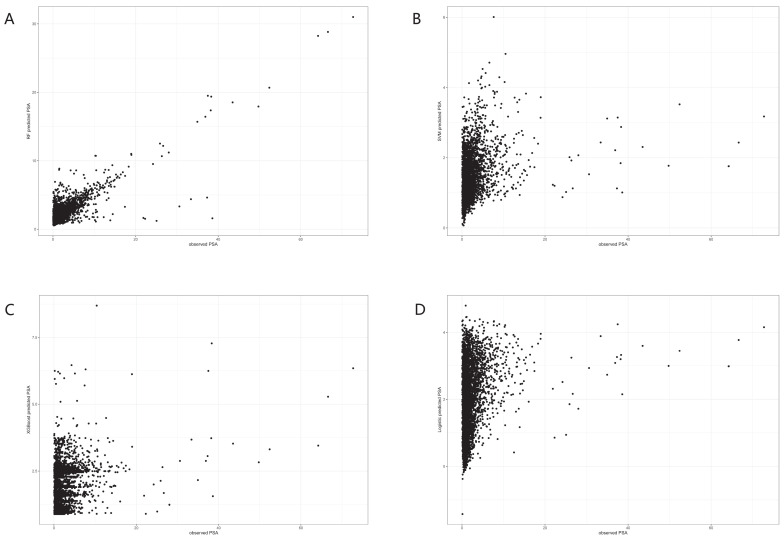
Dot plots for actual and predicted value of PSA in four machine learning algorithms. A. Random forest. B. Logistic Regression. C. XGboost. D. Support vector machine.

In the trained algorithm, we input 25(OH)D and all the covariates incorporated in this paper into the algorithm to obtain the predicted PSA and made a scatter plot with the actual PSA value as the horizontal coordinate and the predicted value as the vertical coordinate. Figure 4A showed the scatter plot of the true and predicted values of the random forest, which could be seen to be roughly linear, indicating that RF had good effects at predicting PSA levels. Figures 4B-D did not show a linear trend.

## Discussion

In this investigation, we employed stepwise logistic regression to scrutinize the relationship between 25(OH)D and PSA levels. The Odds Ratio (OR) of Quartile 4 (Q4) exhibited a significant elevation compared to Q2 and Q3 across different covariates, with a statistically significant trend (p for trend). These findings strongly indicate a positive correlation, signifying that 25(OH)D influences PSA levels in a manner consistent and stable. The detailed results highlighted an OR of Q4 well beyond 1, suggesting a potentially harmful impact of elevated 25(OH)D levels on PSA, particularly in instances of high 25(OH)D concentrations.

Recent clinical trials, such as those conducted by Schwartz *et al.* 18 and Morris *et al.* 19, failed to demonstrate a significant response to 25(OH)D when administered in combination with chemotherapy. Notably, a study led by Srinivas *et al.* 20 was discontinued due to a higher mortality rate in the 25(OH)D supplementation group compared to the placebo group. This observation raises concerns about a plausible association between high 25(OH)D levels and an elevated risk of prostate cancer.

In contrast, OR values in Q2 and Q3 groups were consistently below 1 in both Model 1 and Model 3, suggesting a potential protective effect of 25(OH)D on PSA at lower 25(OH)D levels. Cholesterol-synthesizing species have been reported to produce 25(OH)D3, with its metabolite 25-dihydroxy25(OH)D3 and the transcription factor 25(OH)D receptor collaborating to regulate gene expression. This intricate process influences hundreds of target genes across various tissues and cell types, initially linked to energy homeostasis but also implicated in energy-demanding innate and adaptive immunity. The observed anticancer effects of 25(OH)D are attributed to its direct control of tumor cell differentiation, proliferation, and apoptosis, along with indirect regulation of immune cells within the malignant microenvironment 21-25.

Our analysis of multiple covariates identified age and BMI as significant factors affecting PSA levels. Higher patient age and increased BMI values were associated with elevated PSA levels, consistent with previous research. Studies, such as the one by Calvocoressi *et al.* 26, have highlighted the frequent occurrence of biologically aggressive prostate cancer in men with advanced age, emphasizing potential implications for treatment decisions. Additionally, a recent review by Wilson *et al.* 27 concluded that obesity is linked to accelerated prostate cancer progression and mortality. In the context of obesity, factors such as the insulin and IGF axis, sex hormone concentrations, and altered adipokine signaling can enhance cancer cell proliferation. Consequently, weight loss strategies are posited to offer substantial benefits for obese prostate cancer patients.

Further analysis of the relationship between 25(OH)D and PSA involved Restricted Cubic Spline (RCS) analysis, revealing a U-shaped relationship with a modest slope. Results indicated that circulating 25(OH)D decreases PSA in the range of 20-134 ng/mL, with a potential increase beyond this range, particularly after 134 ng/mL. Discrepancies with a meta-analysis, associating circulating 25(OH)D levels greater than 37.5 nmol/L (15 ng/mL) with an increased risk of aggressive prostate cancer, were noted. Despite the differences, these findings contribute to the ongoing exploration of 25(OH)D thresholds.

In addition to elucidating the relationship between 25(OH)D and PSA, our study aimed to develop effective models for predicting PSA levels based on 25(OH)D levels. A comparison of four algorithms - random forest, logistic regression, support vector machine, and XGBoost 16, 30-32 - revealed that the random forest algorithm exhibited superior performance. This determination underscores the suitability of the random forest algorithm for predicting PSA levels and guiding vitamin D dosing.

While our study leveraged the advantages of a large sample size, multiple models for correlation verification, and analysis of nonlinear relationships between 25(OH)D and PSA, certain limitations must be acknowledged. Firstly, the study's reliance on a 2001-2010 epidemiological survey rather than a cohort study limits the ability to establish causation. Secondly, the annual monitoring of blood 25(OH)D and PSA by the laboratory, spanning a decade, may not fully capture continuous 25(OH)D exposure. Finally, the limited number of covariates employed does not eliminate the potential influence of undetected variables, and the exclusion of certain samples due to missing information results in smaller sample sizes.

In conclusion, our study scrutinized the association between 25(OH)D and PSA in 7174 men over a ten-year period (2001-2010), revealing a U-shaped relationship. PSA demonstrated a decline with 25(OH)D in the range of 20-134 ng/mL, potentially increasing above this range, particularly after 134 ng/mL. Additionally, the random forest algorithm proved effective in predicting PSA levels and guiding vitamin D dosing. While the study presents valuable insights, it encourages further research into the intricate relationship between 25(OH). For public health, the intake of vitamin D should be carefully controlled with a reasonable range.

## Funding

This work was supported by National Natural Science Foundation of China (grant number 81673236) and the Young Talent Development Program of Wuhan Fourth Hospital.

## Figures and Tables

**Table 1:** Characteristics of included samples (N=7174).

PSA	Q1(N=1776)	Q2(N=1731)	Q3(N=1835)	Q4(N=1832)	F or Chi-squared value
**Age**					0.001*
	56.1 (11.7)	54.7 (11.6)	60.0 (12.4)	67.2 (11.5)	
**PIR**					0.755
	2.84 (1.64)	2.84 (1.61)	2.75 (1.60)	2.78 (1.58)	
**BMI**					0.005*
Normal (25<)	88 (18.5%)	111 (23.4%)	127 (27.0%)	131 (27.2%)	
Overweight (25≤BMI<30)	185 (38.9%)	175 (36.9%)	146 (31.1%)	144 (29.9%)	
Obese (≥30)	202 (42.5%)	188 (39.7%)	197 (41.9%)	207 (42.9%)	
**Race**					0.007*
Mexican American	77 (16.2%)	108 (22.6%)	103 (21.5%)	61 (12.7%)	
Other Hispanic	31 (6.51%)	31 (6.50%)	26 (5.44%)	24 (5.01%)	
Non-Hispanic White	277 (58.2%)	253 (53.0%)	245 (51.3%)	289 (60.3%)	
Non-Hispanic Black	76 (16.0%)	76 (15.9%)	89 (18.6%)	90 (18.8%)	
Other Race	15 (3.15%)	9 (1.89%)	15 (3.14%)	15 (3.13%)	
**Education**					0.221
Less Than 9th Grade	68 (14.3%)	67 (14.0%)	86 (18.0%)	88 (18.4%)	
9-11th Grade	82 (17.2%)	77 (16.1%)	71 (14.9%)	61 (12.7%)	
High School	120 (25.2%)	112 (23.5%)	106 (22.2%)	115 (24.0%)	
Some College	113 (23.7%)	139 (29.1%)	114 (23.8%)	116 (24.2%)	
College Graduate or above	93 (19.5%)	82 (17.2%)	101 (21.1%)	99 (20.7%)	
**Marital Status**					0.001*
Married	330 (69.3%)	327 (68.6%)	327 (68.4%)	328 (68.5%)	
Widowed	20 (4.20%)	22 (4.61%)	36 (7.53%)	55 (11.5%)	
Divorced	55 (11.6%)	44 (9.22%)	43 (9.00%)	49 (10.2%)	
Separated	10 (2.10%)	15 (3.14%)	13 (2.72%)	13 (2.71%)	
Never married	32 (6.72%)	33 (6.92%)	33 (6.90%)	21 (4.38%)	
Living with partner	29 (6.09%)	36 (7.55%)	26 (5.44%)	12 (2.51%)	
Refused	0 (0.00%)	0 (0.00%)	0 (0.00%)	1 (0.21%)	
**Alcohol**					0.395
Yes	138 (29.0%)	124 (26.0%)	130 (27.2%)	116 (24.2%)	
No	338 (71.0%)	353 (74.0%)	347 (72.6%)	363 (75.8%)	
**Smoking**					0.001*
Yes	312 (65.5%)	302 (63.3%)	323 (67.6%)	323 (67.4%)	
No	164 (34.5%)	174 (36.5%)	155 (32.4%)	155 (32.4%)	
**Hypertension**					0.643
Yes	23 (4.83%)	16 (3.35%)	20 (4.18%)	23 (4.80%)	
No	453 (95.2%)	461 (96.6%)	458 (95.8%)	456 (95.2%)	
**Diabetes**					
Yes	78 (16.4%)	46 (9.64%)	77 (16.1%)	63 (13.2%)	0.001*
No	386 (81.1%)	422 (88.5%)	385 (80.5%)	403 (84.1%)	
Borderline	12 (2.52%)	9 (1.89%)	15 (3.14%)	13 (2.71%)	

*P<0.05

**Table 2:** Association between Serum vitaminD-25(OH)D and PSA in step-wise logistic regressions.

	Model1	Model2	Model3
**Serum vitaminD-25(OH)D**	Reference	Reference	Reference
	-1.39 (-8.29, 6.02)	1.63 (-5.72, 9.54)	0.57 (-6.37, 8.04)
	0.44 (-5.24, 6.46)	1.61 (-4.37, 7.96)	0.26 (-5.94, 6.86)
	6.23 (0.24, 12.57)	7.22 (-0.22, 15.21)	7.73 (0.26, 15.76)
**p trend**	0.007*	0.001*	0.041*

*P<0.05, Model 1= No adjustment, Model 2 = as Model 1 plus adjusted for sex, age (years, continuous), age squared, education (less than high school, high school graduate, some college and above), race (non-Hispanic white, non-Hispanic black, Mexican American, other), self-reported alcohol status (Yes and No) and self-reported smoking status (Yes and No); Model 3 = Model 2 plus adjusted for BMI, self-reported hypertension (Yes and No) and self-reported diabetes (Yes and No).

**Table 3:** Analysis of co-variates influencing PSA based on logistic regression.

Urinary beryllium	OR (95%CI)	P value
**Age**	2.67 (2.24, 3.1)	0.001*
PIR	-1.98 (-5.55, 1.72)	0.29
**BMI**		
Normal (25<)	Reference	
Overweight (25≤BMI<30)	17.52 (7.65, 26.32)	0.001*
Obese (≥30)	10.36 (0.68, 20.18)	0.045*
**Race**		
Mexican American	Reference	
Other Hispanic	-5.18 (-23.92, 18.19)	0.636
Non-Hispanic White	-11.01 (-20.95, 0.19)	0.054
Non-Hispanic Black	10.58 (-4.26, 27.72)	0.172
Other Race	5.23 (-22.17, 42.27)	0.74
**Education**		
Less Than 9th Grade	Reference	
9-11th Grade	5.19 (-13.53, 27.96)	0.613
High School	14.52 (-5.34, 38.55)	0.163
Some College	14.41 (-4.92, 37.67)	0.154
College Graduate or above	22.04 (-0.2, 49.22)	0.052
**Marital Status**		
Married	Reference	
Widowed	-2.05 (-19.03, 18.5)	0.831
Divorced	-7.45 (-19.9, 6.94)	0.294
Separated	26.38 (-0.9, 61.16)	0.059
Never married	1.66 (-17.73, 25.62)	0.879
Living with partner	-5.77 (-18.88, 9.46)	0.437
Refused	320.93 (251.96, 403.41)	0.356
**Alcohol**		
Yes	Reference	
No	6.32 (-3.84, 17.55)	0.232
**Smoking**		
Yes	Reference	
No	1.99 (-7.43, 12.36)	0.69
**Hypertension**		
Yes	Reference	
No	13.22 (-7.93, 39.23)	0.239
**Diabetes**		
Yes	Reference	
No	24.48 (6.45, 45.56)	0.06
Borderline	22.71 (-7.6, 62.96)	0.158

*P<0.05
